# Value of Manchester Acute Coronary Syndromes Decision Rule in the Detection of Acute Coronary Syndrome; a Systematic Review and Meta-Analysis

**Published:** 2018-12-15

**Authors:** Fatemeh Ramezani, Sajjad Ahmadi, Gholamreza Faridaalee, Alireza Baratloo, Mahmoud Yousefifard

**Affiliations:** 1 Physiology Research Center, Faculty of Medicine, Iran University of Medical Sciences, Tehran, Iran; 2 Emergency Medicine Department, Maragheh University of Medical Sciences; Maragheh; Iran.; 3 Department of Health, Maragheh University of Medical Sciences, Maragheh, Iran.; 4 Emergency Department, Sina Hospital, Tehran University of Medical Sciences, Tehran, Iran.

**Keywords:** Acute Coronary Syndrome, Decision Support Techniques, Diagnosis

## Abstract

**Introduction:**

There is still no consensus on the value of Manchester Acute Coronary Syndromes (MACS) decision rule in detecting acute coronary syndrome (ACS). Therefore, the purpose of the present systematic review and meta-analyzes is to summarize the clinical evidence in the evaluation of the value of MACS in the diagnosis of ACS.

**Methods:**

A literature search was performed on the Medline, Embase, Scopus, and Web of Science databases. Outcomes included acute myocardial infarction (AMI) and major adverse cardiac event (MACE). Data were analyzed in the STATA 14.0 statistical program and the results were reported as summary receiver operating characteristics (SROC), sensitivity, specificity, positive and negative likelihood ratio, and diagnostic odds ratio with 95% confidence interval (95% CI).

**Results:**

Finally, 8 articles included in the meta-analysis. The area under the SROC of MACS was excellent in rule out of AMI (AUC = 0.99, 95% CI: 0.97 to 0.99) and MACE (AUC = 0.97, 95% CI: 0.95 to 0.98). The sensitivity and specificity of the troponin-only MACS/history electrocardiogram alone MACS (HE-MACS) in the rule out of AMI were0.99 (95% CI: 0.98-0.99) and 0.22 (95% CI: 0.11-0.37), respectively, and for the original MACS were in order 0.99 (95% CI: 0.98-0.99) and 0.26 (95% CI: 0.20-0.34),. The sensitivity and specificity of the troponin-only MACS / HE-MACS in the rule out of MACE were 0.94 (95% CI: 0.92-0.96) and 0.22 (95% CI: 0.12-0.39) compared to the 0.99 (95% CI: 0.98-0.99) and 0.27 (95% CI: 0.22-0.33) for the original MACS.

**Conclusion:**

The findings of this study showed that original MACS, troponin-only MACS, and HE-MACS are able to rule out AMI and MACE. However, further studies are needed in developing countries to confirm its external validity.

## Introduction

Acute coronary syndrome (ACS) is a leading cause of mortality and disability worldwide. According to the statistics, in 2017, about 18 million deaths from cardiovascular disease have occurred in the world, which has increased by 21.1% over the past 10 years (1). This increase in the burden of cardiovascular disease has led researchers to seek solutions to reduce the incidence of ACS.

Early diagnosis of ACS is one of the most effective methods to reduce the burden of the disease (2). Currently, the patient’ clinical examination, electrocardiography and serial troponin are the standard ACS diagnostic method. A golden standard for diagnosing ACS in emergency episodes is the serial troponin test (3). However, the long-term approach to diagnosing ACS (about 6-12 hours) is the most important weakness of the current guidelines. In order to overcome this problem, Body et al in 2014 provided the Manchester Acute Coronary Syndromes (MACS) decision rule to the diagnosis of ACS (4). In subsequent studies, the diagnostic value of this model was validated and modifications were made (5-9) (Table 1). In recent years, troponin-only MACS and history and electrocardiogram only MACS (HE-MACS) were introduced to overcome MACS shortcoming. However, there is still no comprehensive overview showing the performance of MACS and its modifications in rule out of ACS. Therefore, the purpose of the current systematic review and meta-analysis is to summarize the clinical evidence in assessing the value of MACS in the diagnosis of ACS.

## Methods

**Search strategy**

In the present meta-analysis using the keywords related to Manchester Acute Coronary Syndromes score in combination with Acute Coronary Syndrome, a search was performed in the Medline, Embase, Scopus, and Web of Science databases. The search was completed by the end of October 2018. No language restrictions were applied. The Searcy query for Medline (via PubMed) has been reported.

1- "Decision Support Techniques"[mh] OR "Manchester Acute Coronary Syndromes"[tiab] OR "MACS prognostic modes"[tiab] OR "MACS prognostic models"[tiab] OR "MACS Rule"[tiab] OR "decision rule"[tiab] OR "Decision Support Techniques"[tiab] OR "Decision Support Technique"[tiab] OR "Technique, Decision Support"[tiab] OR "Techniques, Decision Support"[tiab] OR "Decision Support Technics"[tiab] OR "Decision Support Technic"[tiab] OR "Technic, Decision Support"[tiab] OR "Technics, Decision Support"[tiab] OR "Decision Aids"[tiab] OR "Aid, Decision"[tiab] OR "Aids, Decision"[tiab] OR "Decision Aid"[tiab] OR "Models, Decision Support"[tiab] OR "Decision Support Model"[tiab] OR "Decision Support Models"[tiab] OR "Model, Decision Support"[tiab] OR "Decision Analysis"[tiab] OR "Analyses, Decision"[tiab] OR "Decision Analyses"[tiab] OR "Analysis, Decision"[tiab] OR "Decision Modeling"[tiab] OR "Modeling, Decision"[tiab] OR "Clinical Prediction Rule"[tiab] OR "Clinical Prediction Rules"[tiab] OR "Prediction Rule, Clinical"[tiab] OR "Prediction Rules, Clinical"[tiab] OR "Rule, Clinical Prediction"[tiab] OR "Rules, Clinical Prediction"[tiab]

2- "Acute Coronary Syndrome"[mh] OR "Myocardial Ischemia"[mh] OR "Angina Pectoris"[mh] OR "Angina, Stable"[mh] OR "Angina, Unstable"[mh] OR "Coronary Artery Disease"[mh] OR "Coronary Occlusion"[mh] OR "Coronary Stenosis"[mh] OR "Coronary Thrombosis"[mh] OR "Coronary Vasospasm"[mh] OR "Myocardial Infarction"[mh] OR "Anterior Wall Myocardial Infarction"[mh] OR "Inferior Wall Myocardial Infarction"[mh] OR "Non-ST Elevated Myocardial Infarction"[mh] OR "ST Elevation Myocardial Infarction"[mh] OR "Acute Coronary Syndrome"[tiab] OR "Myocardial Ischemia"[tiab] OR "Angina Pectoris"[tiab] OR "Angina, Stable"[tiab] OR "Angina, Unstable"[tiab] OR "Coronary Artery Disease"[tiab] OR "Coronary Occlusion"[tiab] OR "Coronary Stenosis"[tiab] OR "Coronary Thrombosis"[tiab] OR "Coronary Vasospasm"[tiab] OR "Myocardial Infarction"[tiab] OR "Anterior Wall Myocardial Infarction"[tiab] OR "Inferior Wall Myocardial Infarction"[tiab] OR "Non-ST Elevated Myocardial Infarction"[tiab] OR "ST Elevation Myocardial Infarction"[tiab] OR "Acute Coronary Syndromes"[tiab] OR "Coronary Syndrome, Acute"[tiab] OR "Coronary Syndromes, Acute"[tiab] OR "Syndrome, Acute Coronary"[tiab] OR "Syndromes, Acute Coronary"[tiab] OR "Ischemia, Myocardial"[tiab] OR "Ischemias, Myocardial"[tiab] OR "Myocardial Ischemias"[tiab] OR "Ischemic Heart Disease"[tiab] OR "Heart Disease, Ischemic"[tiab] OR "Disease, Ischemic Heart"[tiab] OR "Diseases, Ischemic Heart"[tiab] OR "Heart Diseases, Ischemic"[tiab] OR "Ischemic Heart Diseases"[tiab] OR "Anginas, Stable"[tiab] OR "Stable Angina"[tiab] OR "Stable Anginas"[tiab] OR "Chronic Stable Angina"[tiab] OR "Angina, Chronic Stable"[tiab] OR "Anginas, Chronic Stable"[tiab] OR "Chronic Stable Anginas"[tiab] OR "Stable Angina, Chronic"[tiab] OR "Stable Anginas, Chronic"[tiab] OR "Angina Pectoris, Stable"[tiab] OR "Angina Pectori, Stable"[tiab] OR "Pectori, Stable Angina"[tiab] OR "Pectoris, Stable Angina"[tiab] OR "Stable Angina Pectori"[tiab] OR "Stable Angina Pectoris"[tiab] OR "Anginas, Unstable"[tiab] OR "Unstable Anginas"[tiab] OR "Angina Pectoris, Unstable"[tiab] OR "Angina Pectori, Unstable"[tiab] OR "Unstable Angina Pectori"[tiab] OR "Unstable Angina Pectoris"[tiab] OR "Unstable Angina"[tiab] OR "Angina at Rest"[tiab] OR "Angina, Preinfarction"[tiab] OR "Anginas, Preinfarction"[tiab] OR "Preinfarction Angina"[tiab] OR "Preinfarction Anginas"[tiab] OR "Myocardial Preinfarction Syndrome"[tiab] OR "Myocardial Preinfarction Syndromes"[tiab] OR "Preinfarction Syndrome, Myocardial"[tiab] OR "Preinfarction Syndromes, Myocardial"[tiab] OR "Syndrome, Myocardial Preinfarction"[tiab] OR "Syndromes, Myocardial Preinfarction"[tiab] OR "Artery Disease, Coronary"[tiab] OR "Artery Diseases, Coronary"[tiab] OR "Coronary Artery Diseases"[tiab] OR "Disease, Coronary Artery"[tiab] OR "Diseases, Coronary Artery"[tiab] OR "Coronary Arteriosclerosis"[tiab] OR "Arterioscleroses, Coronary"[tiab] OR "Coronary Arterioscleroses"[tiab] OR "Atherosclerosis, Coronary"[tiab] OR "Atheroscleroses, Coronary"[tiab] OR "Coronary Atheroscleroses"[tiab] OR "Coronary Atherosclerosis"[tiab] OR "Arteriosclerosis, Coronary"[tiab] OR "Coronary Occlusions"[tiab] OR "Occlusion, Coronary"[tiab] OR "Occlusions, Coronary"[tiab] OR "Stenoses, Coronary"[tiab] OR "Stenosis, Coronary"[tiab] OR "Coronary Artery Stenosis"[tiab] OR "Artery Stenoses, Coronary"[tiab] OR "Artery Stenosis, Coronary"[tiab] OR "Coronary Artery Stenoses"[tiab] OR "Stenoses, Coronary Artery"[tiab] OR "Stenosis, Coronary Artery"[tiab] OR "Coronary Stenoses"[tiab] OR "Infarction, Myocardial"[tiab] OR "Infarctions, Myocardial"[tiab] OR "Myocardial Infarctions"[tiab] OR "Cardiovascular Stroke"[tiab] OR "Cardiovascular Strokes"[tiab] OR "Stroke, Cardiovascular"[tiab] OR "Strokes, Cardiovascular"[tiab] OR "Heart Attack"[tiab] OR "Heart Attacks"[tiab] OR "Myocardial Infarct"[tiab] OR "Infarct, Myocardial"[tiab] OR "Infarcts, Myocardial"[tiab] OR "Myocardial Infarcts"[tiab]

3- #1 and #2

**Selection criteria**

Inclusion criteria included relevant studies in the assessment of MACS in rule out of acute myocardial infarction (AMI) and major acute cardiac event (MACE). Exclusion criteria included the lack of the required data after contact with the authors, duplicate articles, and reviews.

**Data synthases and quality control**

The method of summarizing and collecting data has been reported by previous systematic reviews (10-25). In summary, two independent reviewers screened the titles and abstracts as well as full text of the articles and selected relevant studies. Any disagreement was resolved by discussion with the third reviewer.

The collected data included the name of the first author , the year of study, the number of patients (normal, AMI, and MACE), the type of study (derivation and validation), the type of MACS (the original MACS, the troponin-only MACs, and HE-MACS), the assessed outcome (AMI and MACE) and follow-up duration. MACE included all-cause mortality, AMI, and urgent coronary revascularization. Quality assessment of the articles was evaluated using the proposed method of Quality Assessment of Diagnostic Accuracy Studies 2 (QUADAS-2) guideline (26).

**Statistical analysis**

All analyzes were performed in the STATA 14.0 statistical program. Using the values reported in the relevant studies, we calculated true positive, true negative, false positive and false negative. Data were analyzed by “midas” command and the results were reported as a summary receiver operating characteristic (SROC), sensitivity, specificity, positive and negative likelihood ratio, and diagnostic odds ratio with 95% confidence interval (95% CI). The publication bias was evaluated using Deeks' funnel plot asymmetry test. In all analyzes p <0.05 was considered a significant level.

## Results

**Characteristics**

**Figure 1:**
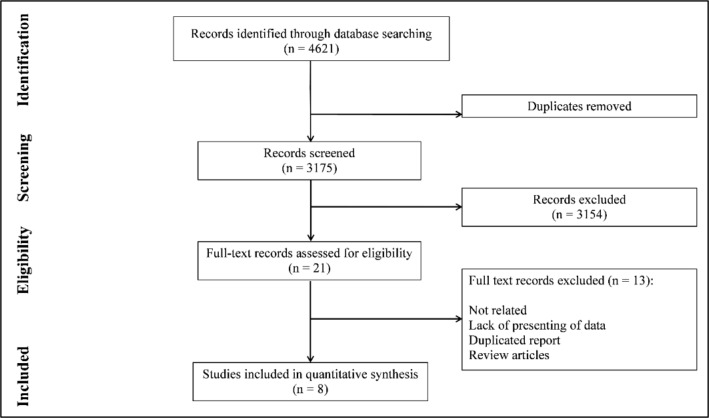
Flow diagram of present meta-analysis

The search led to the achievement of 3175 non-repetitive records. After the initial screening, 21 articles were studied in detail, and finally, eight papers arrived at the quantitative analysis (4-7, 9, 27-29). Figure 1 illustrates the flow diagram of the present meta-analysis. These eight articles contained 33 separate experiments. There were six cohort articles and 2 of them were observational trials. Data from 7658 patients were analyzed. Of these, there were 1203 AMI and 1504 MACE. 64.29% of patients were male. Two studies were derivation-validation and other was validation. Table 2 provides a summary of related articles.

**Quality control of articles and risk of bias**

**Figure 2:**
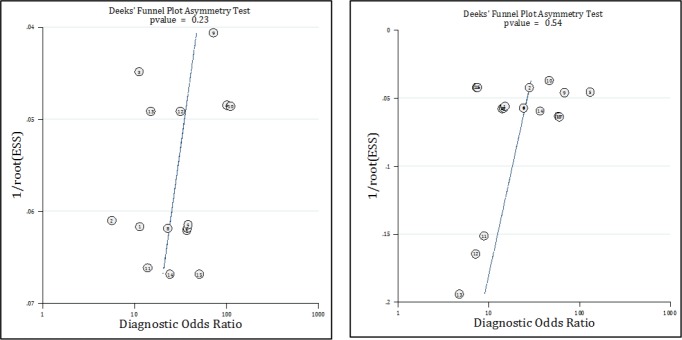
Publication bias in assessment of value of Manchester Acute Coronary Syndromes score in prediction of acute myocardial infarction (A) and major adverse cardiac events (B).

The quality assessment showed that the applicability of patient selection was high risk and unclear in two studies. In addition, in one study, the risk of bias in patient selection was unclear. In other cases, the risk of bias were low. Table 3 shows the quality status of the articles. Deeks funnel plot asymmetry test showed that there was no publication bias in the evaluation of the MACS in the rule out of AMI and MACE (Figure 2).

**The value of MACS in rule out of AMI**

**Figure 3:**
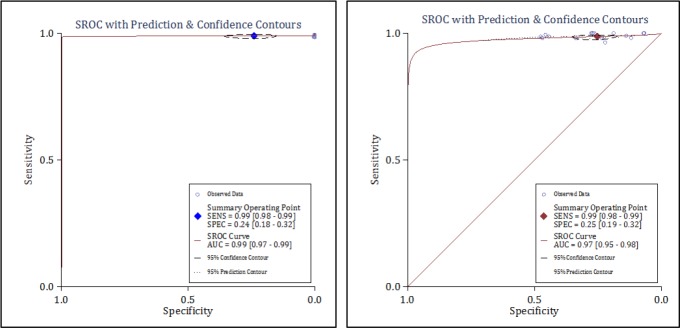
Summary receiver operator characteristics curve of Manchester Acute Coronary Syndromes score in prediction of acute myocardial infarction (A) and major adverse cardiac events (B).

**Figure 4:**
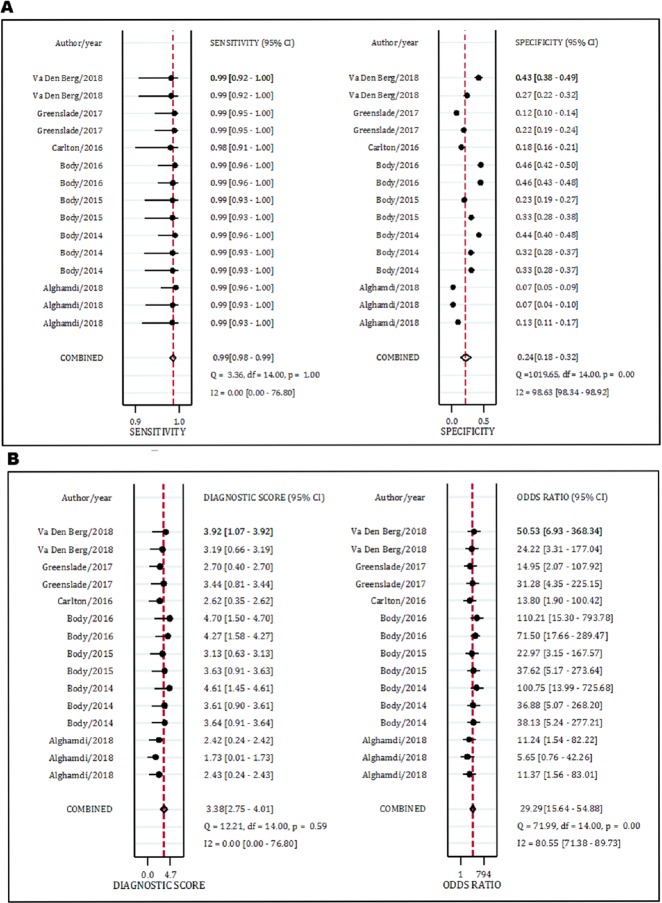
Performance of Manchester Acute Coronary Syndromes score in prediction of acute myocardial infarction and major adverse cardiac events.

Data from seven studies (15 separate experiments) included in this section. The analysis showed that the area under the SROC curve of MACS in the rule out of AMI was excellent (AUC = 0.99, 95% CI: 0.97 to 0.99) (Figure 3). The sensitivity and specificity of the MACS decision aid in the diagnosis of AMI were 0.99 (95% CI: 0.98-0.99) and 0.24 (95% CI: 0.18-0.32), respectively. In addition, DOR of this rule out criteria was 29.0 (95% CI: 16.0-55.0) (Figure 4 and Table 4).

The sensitivity and specificity of troponin-only MACS / HE-MACS in the diagnosis of AMI was 0.99 (95% CI: 0.98-0.99) and 0.22 (95% CI: 0.11-0.37), respectively. These values for the original MACS were 0.99 (95% CI: 0.98-0.99) and 0.26 (95% CI: 0.20-0.34), respectively. In thesensitivity (0.99 vs. 0.99) and specificity (0.26 vs. 0.24) of MACS there was no differencebetween validation and derivation studies (Table 4).

**Diagnostic value of MACS in MACE detection**

**Figure 5:**
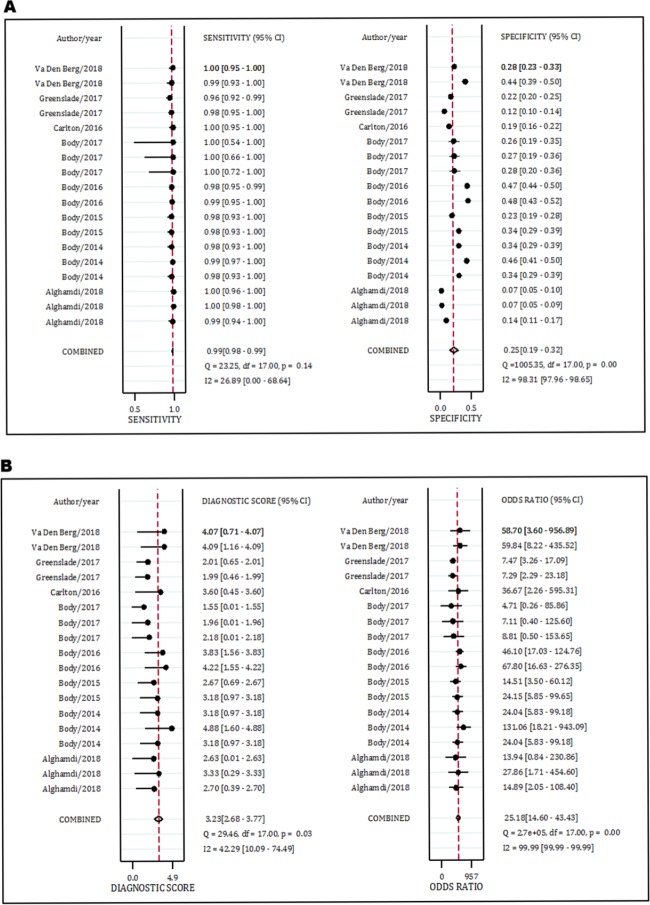
Performance of Manchester Acute Coronary Syndromes score in prediction of major adverse cardiac events.

Data from eight studies (18 separate experiments) were included in this section. The analysis showed that the area under the SROC curve of MACS in the rule out of MACE was excellent (AUC = 0.97, 95% CI: 0.95 to 0.98) (Figure 3). The sensitivity, specificity and DOR of the MACS rule in prediction of MACE were 0.99 (95% CI: 0.98-0.99) and 0.25 (95% CI: 0.19-0.32), and 25.0 (95% CI: 13.0-43.0), respectively (Figure 5 and Table 4).

The sensitivity and specificity of the troponin-only MACS / HE-MACS in the rule out of MACE was 0.94 (95% CI: 0.92-0.96) and 0.22 (95% CI: 0.12-0.39), respectively. These values for the original MACS were 0.99 (95% CI: 0.98-0.99) and 0.27 (95% CI: 0.22-0.33) respectively. The sensitivity (0.98 vs. 0.97) and specificity (0.27 vs. 0.25) of MACS were not different in the MACE prediction between the validation and derivation studies (Table 4). 

## Discussion:

The findings of this study showed that the MACS value is good in the screening of AMI and MACE. It was also found that the value of troponin-only / HE-MACS is similar to the original MACS. Since troponin-only MACS and HE-MACS have fewer variables and don’t need to evaluate the heart-type fatty acid binding protein, it seems that the use of troponin-only / HE-MACS is better than the original MACS.

**Table 1:** Manchester Acute Coronary Syndromes score (MACS)

	**Original MACS**	**Troponin-only MACS**	**HE-MACS**
Heart-type fatty acid binding protein	**✓**	-----	-----
High sensitivity troponin T	**✓**	**✓**	-----
ECG ischaemia	**✓**	**✓**	**✓**
Sweating observed	**✓**	**✓**	**✓**
Vomiting	**✓**	**✓**	**✓**
Systolic blood pressure <100 mmHg	**✓**	**✓**	**✓**
Worsening angina	**✓**	**✓**	-----
Pain radiating to right arm or shoulder	**✓**	**✓**	**✓**
Current tobacco smoker	-----	-----	**✓**
Age	-----	-----	**✓**
Male sex	-----	-----	**✓**

**Table 2:** characteristics of included studies

**Author; year; country**	**Age group**	**Sample size**	**Male gender**	**Type of study**	**Timing of blood sample**	**Sampling**	**Type of MACS**	**Outcome**	**Follow-up (day)**
Alghamdi; 2018; UK	Adult	1902	1160	Derivation-validation cohort	0	Consecutive	HE-MACS	MACE	30
Body; 2014; UK	Adult	1161	699	Derivation-validation cohort	0	Consecutive	Original MACS and Tro-only	AMI; MACE	30
Body; 2015; UK	Adult	456	264	Validation cohort	0	Consecutive	Original MACS	AMI; MACE	30
Body; 2016; UK	Adult	1577	1304	Validation cohort	0	Consecutive	Tro-only	AMI; MACE	30
Body; 2017; UK	Adult	131	79	Validation trial	0	Consecutive	Original MACS	MACE	30-90
Carlton; 2016; UK	Adult	782	466	Validation cohort	0	Consecutive	Original MACS	AMI; MACE	30
Greenslade; 2017; Australia/New Zealand	Adult	1244	719	Validation trial/cohort	0	Unclear	Original MACS and Tro-only	AMI; MACE	30
Va Den Berg; 2018; UK	Adult	405	233	Validation cohort	0	Consecutive	Original MACS and Tro-only	AMI; MACE	30

AMI: Acute myocardial infarction; h-FABP: heart type fatty acid binding protein; HE-MACS: History and electrocardiogram-only Manchester Acute Coronary Syndromes score; MACE: Major adverse cardiac events; MACS: Manchester Acute Coronary Syndromes score; Tro-only: Troponin-only.

**Table 3:** Quality assessment of included articles

**Author**	**Risk of bias**					**Applicability**		
	**Patient selection**	**Index test**	**Reference standard**	**Flow and timing**		**Patient selection**	**Index test**	**Reference standard**
**Alghamdi; 2018**	Low risk	Low risk	Low risk	Low risk		Low risk	Low risk	Low risk
**Body; 2014**	Low risk	Low risk	Low risk	Low risk		Low risk	Low risk	Low risk
**Body; 2015**	Low risk	Low risk	Low risk	Low risk		Low risk	Low risk	Low risk
**Body; 2016**	Low risk	Low risk	Low risk	Low risk		Low risk	Low risk	Low risk
**Body; 2017**	Low risk	Low risk	Low risk	Low risk		Low risk	Low risk	Low risk
**Carlton; 2016**	Low risk	Low risk	Low risk	Low risk		Low risk	Low risk	Low risk
**Greenslade; 2017**	Low risk	Low risk	Low risk	Low risk		**High risk**	Low risk	Low risk
**Va Den Berg; 2018**	**Unclear**	Low risk	Low risk	Low risk		**Unclear**	Low risk	Low risk

**Table 4:** Performance of MACS in detection of cardiac events among subgroups

**Subgroup**	**Number of experiments**	**AUC** **(95% CI)**	**Sensitivity** **(95% CI)**	**Specificity** **(95% CI)**	**PLR** **(95% CI)**	**NLR** **(95% CI)**	**DOR** **(95% CI)**
**AMI**							
**Overall**	15	0.99 (0.97-0.99)	0.99 (0.98-0.99)	0.24 (0.18-0.32)	1.3 (1.2-1.4)	0.04 (0.03-0.08)	29.0 (16.0-55.0)
**Type of MACS**							
Original MACS	8	0.99 (0.97-0.99)	0.99 (0.98-0.99)	0.26 (0.20-0.34)	1.3 (1.1-1.5)	0.02 (0.008-0.07)	31.0 (14.0-68.0)
Tro-only and HE-MACS	7	0.99 (0.97-0.99)	0.99 (0.98-0.99)	0.22 (0.11-0.37)	1.5 (1.3-1.7)	0.03 (0.01-0.07)	51.0 (16.0-162.0)
**Type of study**							
Validation	4	0.99 (0.98-1.0)	0.99 (0.98-1.0)	0.26 (0.12-0.48)	1.3 (1.0-1.7)	0.03 (0.01-0.10)	42.0 (11.0-163.0)
Derivation	11	0.99 (0.97-0.99)	0.99 (0.98-0.99)	0.24 (0.17-0.32)	1.3 (1.2-1.4)	0.05 (0.03-0.10)	25.0 (13.0-51.0)
**MACE**							
**Overall**	18	0.97 (0.95-0.98)	0.99 (0.98-0.99)	0.25 (0.19-0.32)	1.3 (1.2-1.4)	0.05 (0.03-0.09)	25.0 (13.0-43.0)
**Type of MACS**							
Original MACS	11	0.98 (0.97-0.99)	0.99 (0.98-0.99)	0.27 (0.22-0.33)	1.4 (1.3-1.5)	0.05 (0.03-0.09)	27.0 (14.0-53.0)
Tro-only and HE-MACS	7	0.94 (0.92-0.96)	0.99 (0.97-1.0)	0.22 (0.12-0.39)	1.3 (1.1-1.5)	0.05 (0.02-0.12)	27.0 (10.0-71.0)
**Type of study**							
Validation	4	0.98 (0.96-0.99)	0.99 (0.98-1.0)	0.27 (0.12-0.49)	1.4 (1.0-1.8)	0.03 (0.01-0.08)	48.0 (16.0-146.0)
Derivation	14	0.97 (0.95-0.99)	0.98 (0.97-0.99)	0.25 (0.19-0.32)	1.3 (1.2-1.4)	0.06 (0.04-0.10)	20.0 (12.0-33.0)

AMI: Acute myocardial infarction; CI: Confidence interval; AUC: Area under the curve; DOR: Diagnostic odds ratio; HE-MACS: History and electrocardiogram-only MACS; MACE: Major adverse cardiac events; MACS: Manchester Acute Coronary Syndromes score; NLR: Negative likelihood ratio; PLR: Positive likelihood ratio; Tro-only: Troponin-only

The MACS sensitivity to rule out of ACS is very high, but its specificity is low. Therefore, MACS is a good screening tool for AMI diagnosis and MACE prediction. In line with the present review article, Reynard et al. by reviewing five studiesconcluded that MACS is a good model for rule out of ACS (26).

In the present study, 8 articles included in the meta-analysis. Although data from 7658 patients were analyzed, 7 studies were conducted by a team in the UK (4-7, 9, 27, 28). Only one study in Australia and New Zealand performed , that only 405 patients were examined (29). Therefore, external validation of MACS in other communities is also required to ensure it is generalized.

The findings of this study indicate that the performance of troponin-only MACS or HE-MACS in rule out of ACS is not different from the original MACS. One of the main disadvantages of the original MACS is the need to evaluate the heart-type fatty acid binding protein (h-FABP), while in the other two models, it is not necessary to measure h-FABP . The value of the troponin-only MACS in rule out of ACS was evaluated in four studies, while the value of HE-MACS was examined only in one study. Since the HE-MACS method does not require measurement of high sensitivity troponin and h-FABP, so if the screening value of HE-MACS is confirmed, it will be easier to use for rule out the ACS. Further studies are needed to achieve this goal.

## Conclusion:

The findings of this study showed that the original MACS, troponin-only MACS, and HE-MACS are capable to rule out AMI and MACE. However, further studies are needed in developing countries for external validation.
